# Risk of mortality associated with respiratory syncytial virus and influenza infection in adults

**DOI:** 10.1186/s12879-017-2897-4

**Published:** 2017-12-20

**Authors:** Yong Shik Kwon, Sun Hyo Park, Mi-Ae Kim, Hyun Jung Kim, Jae Seok Park, Mi Young Lee, Choong Won Lee, Sonila Dauti, Won-Il Choi

**Affiliations:** 1 Department of Internal Medicine, Keimyung University Dongsan Hospital, Daegu, Republic of Korea; 2 Department of Preventive Medicine, Keimyung University School of Medicine, Dongsan Hospital, Daegu, Republic of Korea; 3 Department of Occupational & Environmental Medicine, Sungso Hospital, Andong, Republic of Korea; 4 Department of Allergology, Hospital Serive of Kavaje, Kavaje, Albania; 5 Department of Internal Medicine, Keimyung University School of Medicine, 194 Dongsan-Dong, Jung-Gu, Daegu, 700-712 Korea

**Keywords:** Respiratory syncytial viruses, Influenza, human, Mortality

## Abstract

**Background:**

Respiratory syncytial virus (RSV) infection constitutes a substantial disease burden in the general population. However, the risk of death for RSV infection has been rarely evaluated with confounders or comorbidities adjusted. We aimed to evaluate whether RSV infection is associated with higher mortality than seasonal influenza after adjusting for confounders and comorbidities and the effect of oseltamivir on the mortality in patients with influenza infection.

**Methods:**

A retrospective cohort study was conducted on adult (≥18 years) patients admitted to the emergency department and ward of a university teaching hospital for suspected viral infection during 2013–2015 (*N* = 3743). RSV infection was diagnosed by multiplex PCR (*N* = 87). Adults hospitalized for seasonal influenza during the study period were enrolled as a comparison group (*n* = 312). The main outcome was 20-day all-cause mortality.We used Cox proportional hazard regression analyses to calculate the relative risk of death.

**Results:**

Adult patients were less likely to be diagnosed with RSV than with influenza (2.3 vs 8.3%, respectively), were older and more likely to be diagnosed with pneumonia, chronic obstructive pulmonary disease, hypoxemia, and bacterial co-infection. In patients with RSV infection, the 20-day all-cause mortality was higher than that for influenza, (18.4 vs 6.7%, respectively). RSV infection showed significantly higher risk of death compared to the seasonal influenza group, with hazard ratio, 2.32 (95% CI, 1.17–4.58). Oseltamivir had no significant effect on mortality in patients with influenza.

**Conclusions:**

RSV infection was significantly associated with a higher risk of death than seasonal influenza, adjusted for potential confounders and comorbidities.

## Background

Respiratory syncytial virus (RSV) is a significant cause of lower respiratory tract infections in infants, and usually requires hospitalization, with the occurrence of death in extreme cases [1, 2].

During the past decades, many studies have clearly demonstrated RSV as a serious pathogen in certain adult populations including long-term care facilities [3–6] and immunocompromised host [7–10]. Although RSV infection in adults is often mild, the effects of RSV infection can be substantial in both healthy adults and hospitalized patients [11]. RSV infection can cause significant morbidity in hospitalized patients, with mortality rates up to 12% [12]. A recent study showed that hospitalized community-acquired pneumonia related with viral pathogens, influenza, and RSV comprised of 21% and 11% among all viral pathogens, respectively [13].

A retrospective cohort study showed that RSV infection, in combination with bacterial co-infection was associated with increased risk of mortality [14]. RSV infections may contribute to 5% to 10% of cases of chronic obstructive pulmonary disease (COPD) exacerbations [15], and 7% of asthma admissions [11] in adults. Virus infections including RSV may explain seasonal mortality in the elderly [16].

In the 1990’s, Influenza had attributable mortality more than 4 times higher than RSV in the elderly in a population-based study [17]. However, in the 2000’s attributable mortality of influenza was less than 2 times compared with those of RSV [18]. Attributable hospitalization due to RSV was almost two-thirds those of influenza [19]. RSV is an increasingly common cause of illness and high morbidity in adults. A better understanding of complications and outcomes of adults hospitalized with RSV compared to influenza infection would be useful to identify high-risk adults. Furthermore, oseltamivir has been commonly prescribed in influenza infected patients since 2009. However, the efficacies of oseltamivir treatment are contradictory [20–25].

We investigated the 20-day all-cause mortality rate for hospital admitted patients with laboratory confirmed RSV compared to influenza. We also investigated the effect of oseltamivir on the mortality of patients with influenza infection.

## Methods

### Study population

**Fig. 1:**
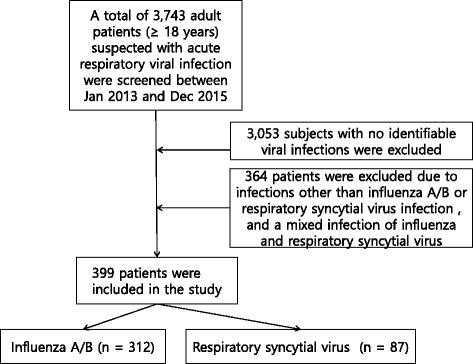
Flow chart of patients suffering from respiratory virus infection

A retrospective cohort study of hospitalized adults with RSV infection was conducted. All patients aged ≥18 years admitted between January 2013 and December 2015, with suspected respiratory viral infections (*N* = 3743), were studied. If physicians suspected respiratory viral illness, then virus multiplex PCR was performed. We excluded 2980 subjects who had no identifiable viral infections, and 364 patients with viral infections other than influenza A/B or RSV infection (Fig. 1). RSV patients (*n* = 87) were compared with patients diagnosed with seasonal influenza (*n* = 312) during the same period (2013–2015).One patient had a mixed infection of influenza and RSV. Among RSV-infected patients, one patient had a mixed infection with bocavirus. Among influenza-infected patients, 5 patients had mixed infection with bocavirus, and 3 patients with coronavirus. All the patients with mixed infections were included in this study except the one patient who had a mixed infection of influenza and RSV.

The current study was approved by the institutional review board at Dongsan Hospital, Keimyung University School of Medicine. The need for written informed consent was waived. This study was conducted in compliance with the Declaration of Helsinki.

### Definitions

An upper respiratory infection was defined as the presence of one or more of the following respiratory symptoms: cough, sputum production, rhinorrhea, sore throat, or dyspnea. Pneumonia was defined as the presence of a new or progressive infiltrate found using chest radiography, in addition to two or more of the following: fever, sputum production, rhinorrhea, sore throat, dyspnea, or a diagnosis of pneumonia by the attending physician. The outcome was designated as all-cause mortality up to 20 days after hospital admission.

### Specimens

During the study period, nasopharyngeal specimens were obtained using flocked swabs and stored and transported using the universal transport medium (COPAN, Brescia, Italy). Nasopharyngeal specimens were submitted for respiratory virus detection. Nucleic acids were extracted from 300 μL specimens using a Viral Gene-spin™ Viral DNA/RNA Extraction Kit (iNtRON Biotechnology, Seongnam, Korea). cDNA was synthesized from each of the extracted RNA samples with cDNA Synthesis Premix (Seegene, Seoul, Korea) and a GeneAmp PCR System 9700 thermal cycler (Applied Biomaterials, Foster City, CA, USA).

### Respiratory virus testing

Respiratory virus (RV) 16 testing was performed to detect the following viruses: adenovirus, influenza viruses A and B, RSV A, RSV B, parainfluenza viruses 1 to 4, Rhinovirus A/B/C, metapneumovirus, enterovirus, coronavirus 229E, coronavirus NL63, coronavirus OC43, and bocavirus. During the RV16 test, an internal control was added to each specimen to check the entire process from nucleic acid extraction to PCR, according to the manufacturer’s instructions. An Anyplex II RV16 Detection Kit (Seegene, Seoul, Korea) was used to detect 14 types of RNA viruses and two types of DNA viruses, according to the manufacturer’s instructions. Briefly, the assay was conducted in a final volume of 20 μL containing 8 μL of cDNA, 4 μL of 5 × RV primer, 4 μL of 8-methoxypsoralen solution, and 4 μL of 5 × master mix with the CFX96 real-time PCR detection system (Bio-Rad Laboratories Inc., Hercules, CA, USA).

### Data collection

This study was performed at Keimyung University Dongsan Hospital, a 867-bed, tertiary care teaching hospital in Daegu, Republic of Korea. If a patient had an episode of acute respiratory infection at an emergency department or outpatient clinic or within 2 days during admission, he or she underwent multiplex RT-PCR testing. Adult patients (≥ 18 years of age) who underwent multiplex RT-PCR testing between January 2013 and December 2015 were identified by electronic medical records. We collected clinical data using the electronic medical record on general characteristics, co-morbidities, presenting symptoms, lower respiratory complications, requirement of supplemental oxygen therapy and/or ventilatory support, hospitalization duration, and all-cause mortality. Chest radiography was performed on all patients admitted to the hospital, and radiographic interpretation was performed for all RSV and influenza cases by radiologists. Additional laboratory investigations were performed based on the results of a routine blood test. Sputum samples were collected for bacterial culture preparation at admission and during hospitalization. Blood cultures were also performed when indicated. Pneumonia severity index (PSI) consisted four parts, demographics including age and sex, co-morbidity, physical examination, and laboratory findings [26]. PSI score was collected every admitted patient. We contacted patients or their families by phone to identify survival and clinical information if the patients were not followed up regularly.

### Statistical analysis

Baseline characteristics (including age, sex, residency in a long-term care facility, comorbidities, presenting symptoms, and complications) at date of admission for cases and control patients were summarized using descriptive statistics, such as proportion and means (standard deviation, SD). A Chi-squared test was applied for comparison between categorical variables, and two-tailed *t*-tests, for comparison between continuous variables. *P* values <0.05 were considered statistically significant.

Fifty-two patients had missing data on body temperature due to lack of measurement in the outpatient clinic. We imputed 52 missing values for body temperature, 10 for RSV group and 42 for influenza group, using a sequential regression approach [27], assuming that body temperature was missing at random conditioning on all covariates included in the analysis.

The univariate and multivariate Cox regression models were used to evaluate the risk of death from RSV infection compared to that of seasonal influenza. The pneumonia severity index (PSI) score and resident of long-term care facilities were excluded from the final model due to multicollinearity with age. The multicollinearity between adjusting variables was checked by the variance inflation factor (VIF) and the tolerance statistic [28]. The largest VIF was 2.05 for PSI score and average VIF was 1.28. The smallest tolerance was 0.48 for PSI score. However, more detailed collinearity diagnostics indicated collinearity between age and PSI score. And when age and resident of long-term care facilities were entered simultaneously into the model, the hazard ratio for age changed its polarity. We analyzed survival curves for case and control subjects by the Kaplan–Meier method and compared them using the log-rank test. We assessed the assumption of proportional hazards with graphical and goodness-of-fit (GOF) approaches [29]. We considered ties when using Cox proportional hazards with the Breslow method [29]. Adjustments were made for sex, age, chronic obstructive pulmonary disease, body temperature > 37.5 °C, pneumonia, hypoxemia and bacterial superinfection. Selection of covariates for the model building was based on clinical relevance and availability. The candidate variables for adjusting were sex, age, resident of long-term care facilities, COPD, high body temperature, pneumonia, bacterial superinfection, hypoxemia, and PSI score. All statistical analyses were performed using IBM SPSS V.21.0 (SPSS Version 23 for Microsoft Windows, IBM, Armonk, NY).

## Results

### Clinical characteristics

**Fig. 2:**
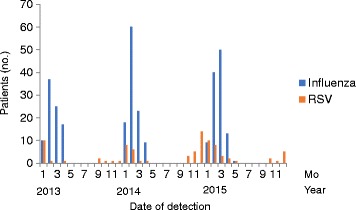
Time of hospital admission of 87 adult patients with virologically confirmed respiratory syncytial virus (RSV) infection, and 312 adult patients with influenza infection, Daegu, Korea, 2013–2015. RSV seasonal peaks occurred during January in 2013, December in 2014, and January in 2015; the percentage test positivity rates during these periods were 11.4% and 40.8% for RSV and influenza, respectively among virus identified patients. Abbreviation: RSV, respiratory syncytial virus

RSV seasonal peaks occurred during January in 2013, December in 2014, and January in 2015 (Fig. 2). The influenza season was defined as period between January and April, and was determined based on the weekly surveillance reports for influenza and other respiratory viruses prepared by the Korea Centers for Disease Control and Prevention.

RSV infection was diagnosed in 87 adult patients (54 A and 33 B), and influenza infection, in 312 adult patients (244 A and 68 B), during the 3-year study period. The percentage test positivity rates during this period were 2.3% and 8.3% for RSV infection and influenza, respectively among all patients tested with respiratory viruses multiplex PCR.

The average age of RSV-infected patients was older than that of influenza patients (70 and 62 years, respectively) (Table 1). RSV patients were more likely to be residents of a long-term care facility or to have COPD, pneumonia, or respiratory bacterial superinfection. Hypoxemia was more frequent and body temperature > 37.5 °C was less common in RSV patients than in influenza patients (Table 1). Oseltamivir was prescribed for 248 influenza patients (79.4%). Mechanical ventilation was used for 3 patients (3.4%) with RSV infection, and for 7 patients (2.2%) with influenza. Mean PSI score was 21 points higher in RSV infection (107.5) than influenza (86.5).

**Table 1:** Baseline characteristics, morbidities, complications, outcomes of respiratory syncytial virus (RSV) and influenza infection cases, 2013-2015

Variables	Influenza A or B (*N* = 312)	RSV (*N* = 87)	*P* value
Male, *n* (%)	153 (49.0)	47 (54.0)	0.41
Age (years) mean (SD)	62.6 (17.1)	70.0 (12.2)	< 0.01
Resident of long-term care facilities, *n* (%)	6 (1.9)	9 (10.3)	< 0.01
Malignancy, n (%)	32 (10.3)	12 (13.8)	0.35
Congestive heart failure, *n* (%)	23 (7.4)	8 (9.2)	0.57
Cerebrovascular accident, *n* (%)	39 (12.5)	11 (12.6)	0.97
Chronic kidney disease, *n* (%)	38 (12.2)	16 (18.4)	0.13
Diabetes, *n* (%)	80 (25.6)	24 (27.6)	0.71
Liver disease, *n* (%)	25 (8.0)	7 (8.0)	0.99
Chronic obstructive pulmonary disease, *n* (%)	15 (4.8)	11 (12.6)	< 0.01
Asthma, *n* (%)	20 (6.4)	3 (3.4)	0.29
Body temperature > 37.5 °C, *n* (%)	110 (35.3)	17 (19.5)	< 0.01
Pneumonia, *n* (%)	78 (25.0)	34 (39.1)	< 0.01
Respiratory bacterial superinfection, *n* (%)	32 (10.3)	16 (18.4)	0.05
Hypoxemia, *n* (%)	93 (29.8)	38 (43.7)	0.01
Mechanical ventilation, *n* (%)	7 (2.2)	3 (3.4)	0.52
PSI score, mean (SD)	86.5 (34.9)	107.5 (30.0)	< 0.01
Twenty-day all-cause mortality (%)	21 (6.7)	16 (18.4)	< 0.01
Sixty-day all-cause mortality (%)	38 (12.2)	22 (25.3)	< 0.01

*PSI* pneumonia severity index, *SD* standard deviation

PSI was calculated in all patients with or without pneumonia

The body temperature was imputed for 52 patients

### Outcomes

**Fig. 3:**
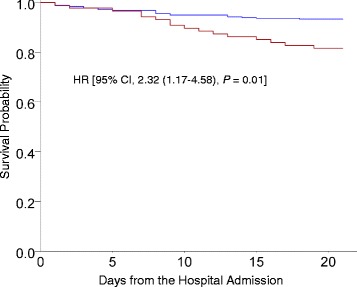
Kaplan-Meier survival curves of 399 adults hospitalized with respiratory syncytial virus (RSV, red line) and influenza infection (blue line). Patients with RSV infection were shown to have lower survival rates. Between RSV and influenza infected patients, the crude 20-day all-cause mortality rate among RSV and influenza infected patients was 18.4% and 6.7%, respectively

All-cause 20-day mortality rates were higher in RSV patients than in influenza patients (18.4 and 6.7%, respectively) (Fig. 3).

After adjusting for potential confounders and comorbidities, the hazard ratio (HR) (2.32; 95% confidence interval [CI], 1.17–4.58) of 20-day all-cause mortality was significantly higher for RSV than influenza (Table 2).

**Table 2:** Variables associated with 20-day all-cause mortality in respiratory syncytial virus and influenza infection

	Univariate analysis		Multivariate analysis	
Variables	HR (95% CI)	*P* value	HR (95% CI)	*P* value
RSV infection	2.83 (1.47-5.43)	< 0.01	2.32 (1.17-4.58)	0.01
Sex	1.88 (0.96-3.70)	0.06	1.87 (0.94-3.73)	0.07
Age	1.02 (0.99-1.04)	0.08	1.00 (0.98-1.03)	0.50
COPD	4.31 (1.97-9.43)	< 0.01	2.97 (1.28-6.90)	0.01
Body temperature (BT) >37.5 °C	0.79 (0.38-1.63)	0.52	1.08 (0.50-2.35)	0.82
Pneumonia	2.00 (1.04-3.84)	0.03	1.72 (0.84-3.51)	0.13
Respiratory Bacterial superinfection	0.89 (0.31-2.52)	0.83	0.56 (0.19-1.65)	0.29
Hypoxemia (< 60 mmHg)	1.79 (0.94-3.43)	0.07	1.05 (0.51-2.18)	0.87

The variables, resident of long-term care facilities and pneumonia severity index score, were not included due to collinearity

*RSV* respiratory syncytial virus, *COPD* chronic obstructive pulmonary disease, *HR* hazard ratio, *CI* confidence interval. Hazard ratios were calculated using a Cox Proportional hazards regression model

### Effects of Oseltamivir in patients with influenza infection

**Fig. 4:**
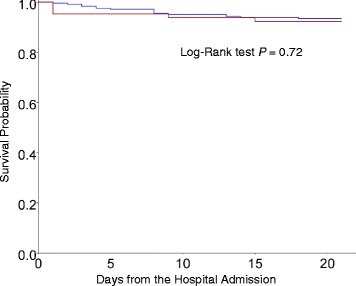
Kaplan-Meier survival curves of 312 adults hospitalized for influenza virus infection, who did not undergo oseltamivir therapy (red line, *n* = 65) and who did undergo oseltamivir therapy (blue line, *n* = 247). Twenty-day all-cause mortality rates among influenza patients treated with and without oseltamivir were 6.4% and 7.6%, respectively (*P* = 0.72)

Eighty percent of influenza virus infected patients were treated with oseltamivir (*n* = 247), while the other 20% of these patients were not prescribed oseltamivir (*n* = 65). They were less likely to be treated with oseltamivir if diabetic, otherwise similar groups (Table 3). Oseltamivir-treated patients had no significant difference in mortality than the patients not treated with oseltamivir did; the 20-day all-cause mortality rates were 6.4% and 7.6%, respectively (*P* = 0.72, Fig. 4).

**Table 3:** Baseline characteristics, morbidities, complications, outcomes of influenza infection cases treat with or without oseltamivir, 2013-2015

Variables	Influenza not treated with Oseltamivir (N = 65)	Influenza treated with Oseltamivir (N = 247)	*P* value
Male, *n* (%)	30 (46.2)	123 (49.8)	0.60
Age (years) mean (SD)	61.2 (18.3)	63.0 (16.7)	0.44
Resident of long-term care facilities, *n* (%)	1 (1.5)	5 (2.0)	0.80
Malignancy, *n* (%)	6 (9.2)	26 (10.5)	0.08
Congestive heart failure, *n* (%)	8 (12.3)	15 (6.1)	0.08
Cerebrovascular accident, *n* (%)	4 (6.2)	35 (14.2)	0.08
Chronic kidney disease, *n* (%)	8 (12.3)	30 (12.1)	0.91
Diabetes, *n* (%)	23 (35.4)	57 (23.1)	0.04
Liver disease, *n* (%)	5 (7.7)	20 (8.1)	0.91
Chronic obstructive pulmonary disease, *n* (%)	6 (9.2)	9 (3.6)	0.06
Asthma, *n* (%)	6 (9.2)	14 (5.7)	0.29
Body temperature > 37.5 °C, *n* (%)	17 (26.2)	93 (37.7)	0.08
Pneumonia, *n* (%)	19 (29.2)	59 (23.9)	0.37
Respiratory bacterial superinfection, *n* (%)	6 (9.2)	26 (10.5)	0.76
Hypoxemia, *n* (%)	17 (26.2)	76 (30.8)	0.47
Mechanical ventilation, *n* (%)	3 (4.6)	4 (1.6)	0.14
PSI score, mean (SD)	85.0 (36.6)	86.9 (34.5)	0.70
Twenty-day all-cause mortality (%)	5 (7.7)	16 (6.5)	0.72
Sixty-day all-cause mortality (%)	12 (18.5)	26 (10.5)	0.09

*PSI* pneumonia severity index, *SD* standard deviation

PSI was calculated in all patients with or without pneumonia

## Discussion

This study showed that all-cause mortality within 20 days after hospital admission was higher in patients with RSV infection (18.4%) than in those with influenza (6.7%) and adjusted hazard ratio (RSV vs. influenza) was 2.32 (95% CI, 1.17–4.58). However, oseltamivir had no significant effect on mortality in patients with influenza.

Previous epidemiologic study and retrospective cohort suggested the mortality rates related to RSV infection in the elderly was estimated 2-17% [12, 30–33]. The varying mortality rates related with RSV infection might be explained by high viral load and severity of infection [32, 33]. In this study, the high PSI score in RSV infection might represent the severity of infection, which may partly explain the higher mortality from RSV infection in the current study than previously reported.

RSV infection is a significant cause of death in infants less than 12 months of age [34, 35]. The serious problem of RSV was underestimated in elderly individuals until the 1970s [36]. Epidemiologic studies revealed that the RSV infection, like influenza infection, is also a significant cause of death or morbidity in elderly individuals [17, 37–40]. However, the risk of mortality with RSV infection in the presence of comorbidities or confounders has not been explored well. In the present study showed RSV infection was an independent risk factor for mortality adjusting variables.

Although RSV data was based on both antigen detection and viral isolation methods [17], only antigen detection tests were used in the surveillance report since 2008 [41]. In addition to RSV, real-time PCR for detection of influenza viruses was implemented in the 2000’s [42–44]. Since real-time PCR is more sensitive than antigen detection or virus isolation [45], there may be a difference in the description of the epidemiology between the pre- and post- PCR time periods. Thus, detecting methods may influence on virus epidemiology. Therefore, it would not be reasonable for direct comparison between previous epidemiologic studies and the present study.

Oseltamivir therapy showed effectiveness on reducing mortality in influenza infection [22–24]. However, there are still controversies about the effect of oseltamivir on the outcome of influenza-infected patients [21, 25]. In the present study, oseltamivir was prescribed in almost 80% of influenza infection cases. Although the difference in mortality rates failed to reach statistical significance, there was a trend towards influenza-infected patients treated with oseltamivir having a lower mortality rate than influenza-infected patients who were not treated with oseltamivir. We suspect that oseltamivir may partly contribute to the difference in mortality rates between RSV and influenza infection cases in the present study.

Our study showed that in adult patients, RSV infections can also cause pneumonia, resulting in respiratory failure in a higher proportion than seasonal influenza. RSV is the most significant cause of lower respiratory tract infections in infants and young children [46] and is known to be a significant cause of severe acute respiratory infections in elderly people [11, 47]. In our study, pneumonia was diagnosed in 39.1% patients. In 1984, similar results were observed in a nursing home for elderly during an outbreak of RSV infection [48].

In the present study, patients with RSV infection exhibited lower body temperature more frequently than patients with influenza did. In 2007, Walsh et al. [49] also reported that RSV infections were more commonly associated with lower temperature than influenza, and a greater proportion of RSV patients developed pneumonia, than influenza patients. Similar results were previously observed in the study from Falsey et al. [39] in 1995 and Dowell et al. [47] in 1996.

The PSI was developed as a clinical prediction tool to guide doctors in deciding between inpatient or outpatient treatment of pneumonia patients [26]. Viral pneumonia may not be detected using simple chest radiography; however, ground glass opacity can be identified using chest computed tomography. Furthermore, RSV and influenza infection may have a significant impact on vital signs, as well as on laboratory findings. In the present study, the PSI score was significantly higher in RSV infection cases than in influenza cases by 21 points. We speculate that RSV infection may result in a more serious inflammatory response than influenza infection.

Although RSV infection accounted for only 2% of all adult respiratory viral infections in the present study, RSV infection should not be regarded as insignificant. If patients are suspected of having RSV infection, a prompt test for the respiratory virus should be the first step towards confirming diagnosis and treating patients. In addition to performing the test, the result should be made available quickly. Identification of the virus, as well as patients who are at a high risk of death, would help in prescribing antiviral therapies appropriately. Recent advances in the development of antiviral therapies for RSV are promising [50, 51], ensuring more options for treating RSV infections in the near future. If an effective vaccine for RSV becomes available, it would be prudent to target high prevalent groups, such as nursing home residents.

This study has several limitations. First, the absence of RSV or influenza in the nasopharynx does not exclude the presence of RSV or influenza in the lower respiratory tract. Thus, the results may underestimate RSV infection or influenza in lower respiratory tract infection. However, it was unlikely to affect the HR estimates. Second, we did not take into consideration vaccination against influenza, which may have reduced the impact of influenza illness. Third, data were retrospectively collected. Missing data and inadequate documentation including time to start medication may have resulted in biases in the study analysis. Fourth, there is a need for validation of the results with extended hospitals because of limited sources of the patients.

## Conclusions

Our study showed that although RSV was detected less frequently in hospitalized adult patients, it was associated with a significantly higher risk of 20-day all-cause mortality than seasonal influenza.

## Abbreviations

CI: Confidence interval
COPD: Chronic obstructive pulmonary disease
HR: Hazard ratio
PSI: Pneumonia severity index
RSV: Respiratory syncytial virus
